# *In Vivo* Optical Imaging of Tumor and Microvascular Response to Ionizing Radiation

**DOI:** 10.1371/journal.pone.0042133

**Published:** 2012-08-22

**Authors:** Azusa Maeda, Michael K. K. Leung, Leigh Conroy, Yonghong Chen, Jiachuan Bu, Patricia E. Lindsay, Shani Mintzberg, Carl Virtanen, Julissa Tsao, Neil A. Winegarden, Yanchun Wang, Lily Morikawa, I. Alex Vitkin, David A. Jaffray, Richard P. Hill, Ralph S. DaCosta

**Affiliations:** 1 Ontario Cancer Institute and Campbell Family Institute for Cancer Research, Princess Margaret Hospital, University Health Network, Toronto, Ontario, Canada; 2 Department of Medical Biophysics, University of Toronto, Toronto, Ontario, Canada; 3 Department of Radiation Oncology, University of Toronto, Toronto, Ontario, Canada; 4 Radiation Medicine Program, STTARR Innovation Centre, Toronto Medical Discovery Tower, Toronto, Ontario, Canada; 5 University Health Network Microarray Centre, Toronto, Ontario, Canada; 6 Centre for Modelling of Human Disease, Mount Sinai Hospital, Toronto, Ontario, Canada; Stanford University, United States of America

## Abstract

Radiotherapy is a widely used cancer treatment. However, understanding how ionizing radiation affects tumor cells and their vasculature, particularly at cellular, subcellular, genetic, and protein levels, has been limited by an inability to visualize the response of these interdependent components within solid tumors over time and *in vivo*. Here we describe a new preclinical experimental platform combining intravital multimodal optical microscopy for cellular-level longitudinal imaging, a small animal x-ray microirradiator for reproducible spatially-localized millimeter-scale irradiations, and laser-capture microdissection of *ex vivo* tissues for transcriptomic profiling. Using this platform, we have developed new methods that exploit the power of optically-enabled microscopic imaging techniques to reveal the important role of the tumor microvasculature in radiation response of tumors. Furthermore, we demonstrate the potential of this preclinical platform to study quantitatively - *with cellular and sub-cellular details* - the spatio-temporal dynamics of the biological response of solid tumors to ionizing radiation *in vivo.*

## Introduction

Radiation therapy is an effective treatment for many cancers and is used in greater than 50% of cancer patients [1]. Ionizing radiation acts by killing tumor cells primarily by damaging their DNA [2]; however, the response of the tumor vasculature and its perivascular support systems are increasingly recognized as critical determinants of biological response of solid tumors, making them attractive targets for improving response to therapy [3]. This is especially true with combination therapies such as anti-angiogenic pharmaceuticals [4]–[6]. Despite the high clinical relevance, studying the complex and interdependent spatial and temporal relationships between irradiation pattern and the tumor cell and vascular response in preclinical tumor models *in vivo* has not been achieved.

Previous efforts to understand radiation response in preclinical solid tumor models have been limited by the lack of ability to study the dynamic response of multiple tumor components (e.g. tumor cells, vasculature and microenvironment) longitudinally and *in situ* at cellular and subcellular scales. Here we present a new methodology that uses a novel preclinical experimental platform to study radiation response of tumors and their vasculature. Using the mouse dorsal skinfold window chamber (DSWC) model, this platform combines a small animal x-ray microirradiator with an intravital multimodal optical microscopy suite comprised of multiplexed fluorescence microscopy and speckle variance optical coherence tomography (svOCT), in a spatiotemporally co-registered manner. We demonstrate the capability and robustness of this experimental platform by investigating the specific biological, structural, and functional response in tumors and surrounding normal tissues following a single-fraction irradiation. Furthermore, *ex vivo* tissue sectioning, followed by laser capture microdissection (LCM) was used to investigate the corresponding radiation-induced transcriptomic modifications in a spatially-registered manner. We have uniquely combined existing optical imaging techniques into a single platform, thereby enabling a new way to quantitatively and longitudinally study radiobiological response in solid tumors *in vivo* while overcoming the limitations of conventional experimental approaches.

## Results

### X-ray Micro-irradiation of Solid Tumors in the Window Chamber

**Figure 1:**
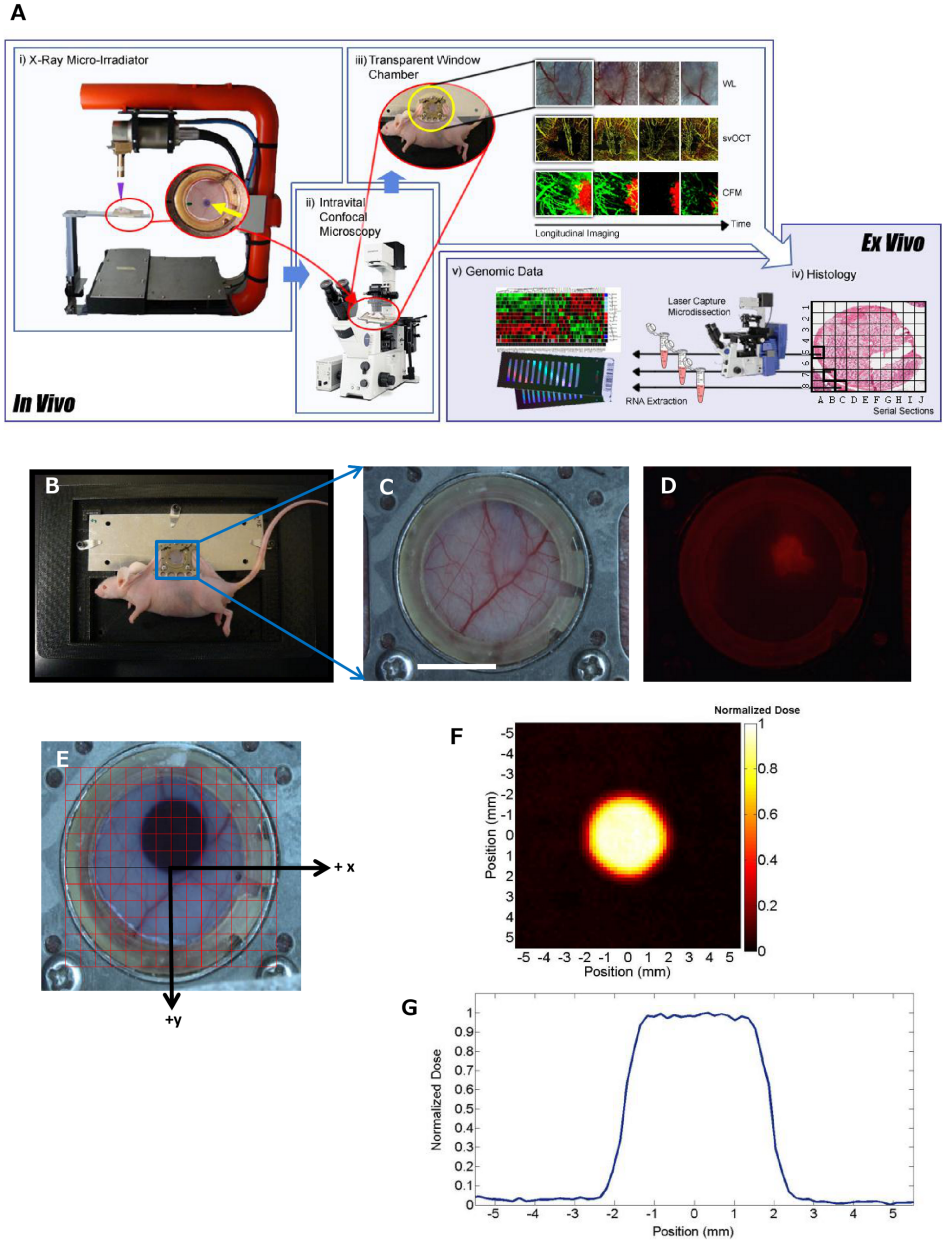
Multimodal technical platform combining *in vivo* optical imaging techniques, small animal x-ray microirradiator and *ex vivo* mRNA analysis. **A.** A schematic diagram and pictures of preclinical platform allowing x-ray micro-irradiation of DSWC tumors, intravital and longitudinal optical imaging (WL: white light, CFM: confocal fluorescence microscopy) and *ex vivo* tissue histological and genomic expression. **B.** Photograph of DSWC mouse with the window chamber mount restrained in a custom made restrainer with a heated adapter. **C.** White light and **D.** corresponding fluorescence images of DsRed-Me180 solid tumor growing subcutaneously. **E.** A radiochromic film was cut to fit the window chamber coverslip diameter for radiation dosimetry and for visualizing the radiation field (4 mm diameter) during optical imaging. The digital grid system (1×1 mm^2^ squares) was centered on the coverslip to facilitate precision-guided delivery of x-ray dose and subsequent co-registration of the intravital optical images and *ex vivo* tissue analysis with tumor and radiation dose positions. **F.** Irradiated film was digitally scanned using a color optical scanner to measure optical density which is directly proportional to radiation dose (color-scale shows the spatial profile of normalized dose (Gy) and **G.** calibrated to quantify the dose (Gy, normalized to the maximum dose). Note the flat profile of the treatment beam across the irradiated tumor area (with 4 mm full-width-at-half-maximum), and the sharp drop off of dose at the boundary of the treatment field. *Scale bar: 5 mm (C).*

DsRed fluorescent Me180 human cervical tumors were grown to a diameter of approximately 3–5 mm in the DSWC. The DsRed fluorescent tumors [7] were focally irradiated using a precision-controlled small animal x-ray micro-irradiator (Figure 1A). A brass collimator with a pinhole diameter of 2.5 mm, yielding an irradiation beam of 4 mm diameter within the DSWC, was used for irradiation of the tumor with 30 Gy. The geometric accuracy of this micro-irradiator has been previously demonstrated [8]. A special plastic restraining device (Figure 1B): i) secured and stabilized the DSWC during micro-irradiation and minimized breathing motion of the anesthetized mouse during imaging, ii) allowed inverted optical microscopy, iii) facilitated transport and experimental reproducibility between the different imaging instruments and the irradiator, and iv) maintained the physiological body temperature of the mouse during irradiation and imaging. A white light and fluorescence photograph of the window chamber with the tumor was taken prior to irradiation (Figure 1C, D). A digital grid (1×1 mm^2^) was used to localize the delivery of the radiation dose to the tumor area and to provide a visual map of tissue and vascular landmarks for subsequent longitudinal optical imaging (Figure 1E). Radiation dosimetry was performed separately using small round pieces of radiochromic film cut to size and placed immediately below the glass coverslip of a window chamber mount. The dose rate was determined to be 2.33 Gy/min with a photon energy of 100 kVp. A highly conformal dose in the irradiation field was achieved with a sharp dose drop-off at the periphery of the field, reducing the out-of-field dose to nearly zero (Figure 1 F, G). Thus, the small animal x-ray micro-irradiator system enabled focal delivery of millimetre-sized x-ray irradiation fields to xenograft tumors implanted in the DSWC. In addition, by using radiochromic film, this technology allowed visualization of the irradiation field boundary as well as accurate radiation dosimetry.

### Simultaneous Imaging of Radiation Response of Tumors and their Vasculature *In Vivo*

**Figure 2:**
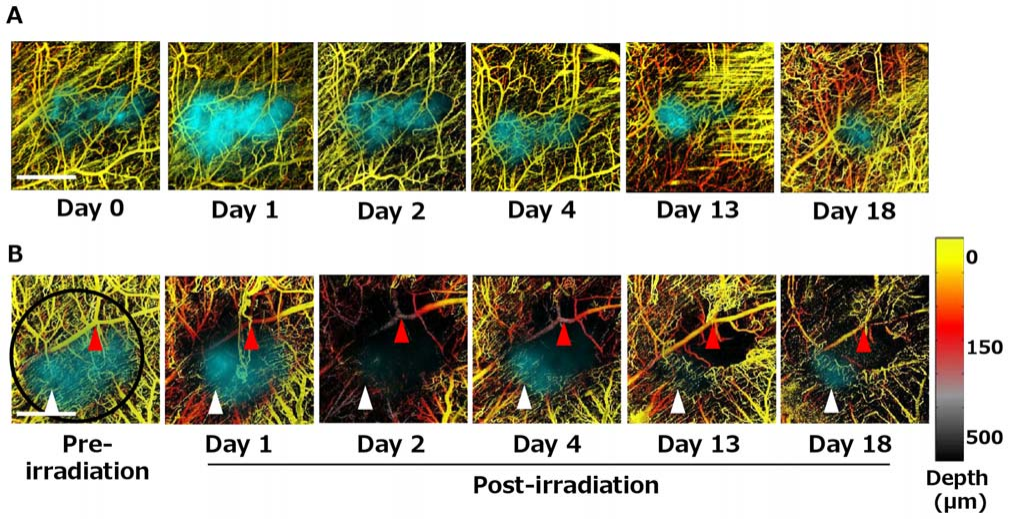
Simultaneous visualization of radiation-induced tumor and microvascular response using longitudinal depth-resolved svOCT microscopy *in vivo*. Comparison of **A.** non-irradiated and **B.** irradiated tumors (30 Gy single fraction with 4 mm diameter treatment spot covering the entire tumor, highlighted in black circle) shows dynamic modification of tumor vasculature after irradiation. DsRed-Me180 tumors are false-colored cyan for better viewing. svOCT data is presented as two-dimensional maximum-intensity projections of a three-dimensional depth-encoded data set. svOCT imaging on day 13 of the non-irradiated mouse showed some motion artifacts, as seen by the horizontal lines. Although motion artifacts were minimized by the use of plastic restraining device, these artefacts could not be completely avoided and were caused by spontaneous muscle spasms. svOCT imaging of the irradiated mouse showed temporary and transient disruption of vascular patency especially in capillaries within the irradiated field. Spatial overlap between residual tumor and functional capillaries remaining within the irradiated field can be observed over the time course (white arrows). In addition, changes in the depth distribution of the vasculature can be observed with this depth-resolved svOCT (red arrows). *Scale bars: 2 mm (A,B).*

We used our imaging platform to observe tumor cellular and vascular radiation responses using wide-field fluorescence microscopy to visualize the DsRed-labeled tumor location and determine tumor size in the DSWC, and svOCT to track the vascular network serially over 18 days following irradiation. The svOCT system had the axial and lateral resolution in tissue of approximately 8 and 13 µm, respectively, which were sufficient to study capillary structures. Figure 2 shows the co-registration and overlay of the DsRed fluorescent tumor (pseudo-colored cyan for better viewing) with the depth-encoded svOCT images of the tumor vasculature (pseudo-colored to indicate depth into the tissue below the coverslip) of both non-irradiated (control) and irradiated tumors. The fluorescence image showed that the tumor remained relatively unchanged over 18 days in a non-irradiated mouse (Figure 2A). On the other hand, Figure 2B illustrates a partial tumor response to irradiation; residual tumors were observed with fluorescence imaging after 18 days, especially in the region where the vasculature remained patent following irradiation (Figure 2B, white arrows). Using svOCT, we observed that the vascular density of the untreated tumor remained relatively constant for the first ∼13 days and then increased as might be expected during tumor-induced angiogenesis [9]. svOCT imaging allowed making comparisons of *in vivo* response to irradiation between vessels of different sizes. As an example, the tumor microvascular density decreased significantly in the irradiated mouse and few capillaries remained visible within the treated area as early as 1 day after irradiation. A significant increase in the density of capillaries and structural remodelling was observed after day 13, despite reduced tumor fluorescence. This observation agrees with previous reports of irradiation-induced angiogenesis in tumors [10]. We noted that larger vessels remained relatively unaffected compared with smaller capillary-sized vessels following irradiation, consistent with previous findings [11]. Depth-resolved svOCT imaging also highlighted the change in the organization of the irradiated vasculature, as indicated by changes in the grey-to-purple false-colored tumor vessels within the irradiated field at days 1, 2 and 4 following irradiation (Figure 2B, red arrows). These results demonstrate that, using intravital fluorescence imaging and svOCT together, it is now possible to visualize tumor response while simultaneously tracking microvascular changes at the structural and functional levels following radiation treatment for approximately 3 weeks *in vivo*.

### Multiparametric Visualization of Radiation Response of Tumors and Vasculature

**Figure 3:**
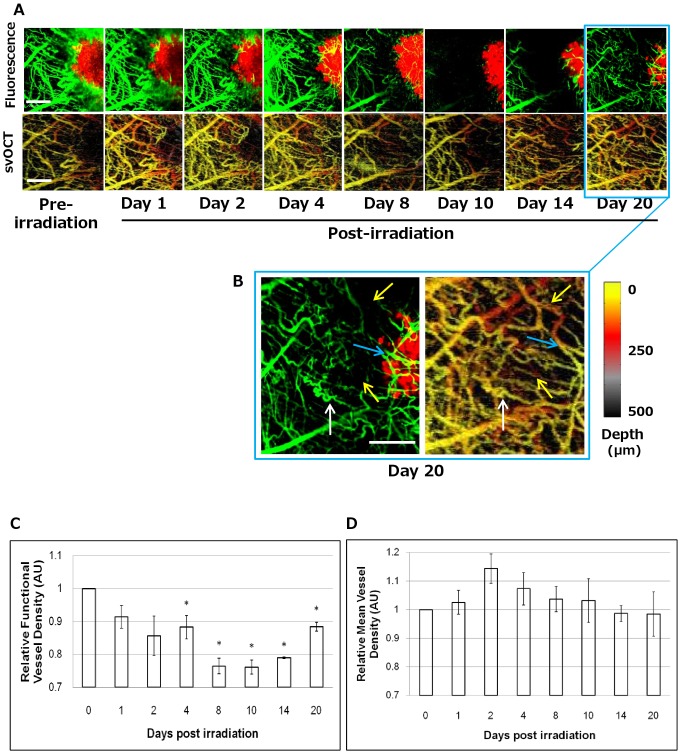
Longitudinal intravital fluorescence and svOCT imaging of radioresponse in tumor and vascular compartments *in vivo*. **A.** Functional changes in perfusion in the tumor microvasculature can be seen over time (FITC-Dextran fluorescence, green) as well as tumor response (DsRed fluorescence, red) following a single fraction of 30 Gy. Image acquisition settings were kept consistent across all the time points. As early as day 4, and maximally at days 8–10 after irradiation, FITC-Dextran signal decreases significantly, indicating capillary constriction resulting in reduction in perfused blood volume. By day 10, there is disruption of blood flow in both large (∼40 µm diameters) and small (∼10 µm diameters) vessels outside and within the tumor. However, the vasculature at these time points is still intact (as seen in svOCT images of corresponding time points). **B.** At day 20 after irradiation, blood vessel perfusion resumes in most vessels around the tumor periphery (*white arrows*), while some vessels appear patent (seen intact in svOCT) without reperfusion as seen in FITC-Dextran (*yellow arrows*), and newly perfused vessels can be observed within the tumor (*blue arrows*). Corresponding svOCT images demonstrate structural changes in the vasculature, particularly close to the tumor margin and within the tumor after irradiation. This was most pronounced at days 8–10. Quantification of **C.** functional vessel density (FITC-Dextran) and **D.** mean vessel density (svOCT), normalized to day 0, of 3 ROI's in irradiated mouse over 20 days. *Scale bars: 400 µm (A,B).*

To address the questions of: i) whether the radiation transiently modified the functional integrity of the small vessels or damaged the vessel structure and ii) whether the vascular changes were spatiotemporally specific (i.e. does proximity of the vessels to the tumor have an effect on radiosensitivity), we used intravital multichannel confocal fluorescence microscopy to image the DsRed-Me180 tumor cells as well as FITC-Dextran-labeled vasculature repeatedly over 20 days following irradiation. FITC-Dextran imaging provided high resolution morphological and functional information based on blood perfusion of the fluorescent Dextran agent, while svOCT images provided spatiotemporally correlated maps of patent vasculature as svOCT is capable of imaging vessels provided they contain blood regardless of flow [12]. The two complementary modalities were able to achieve a similar spatial resolution, enabling imaging of capillaries with average diameters in the range of 10 µm. Figure 3A shows the response of a small region-of-interest within the tumor area demonstrating gradual and significant decrease in vascular perfusion beginning as early as day 2, and subsequent reperfusion after day 14. The volume and concentration of FITC-Dextran injected into the mice, as well as the timing of imaging following its administration were kept consistent across each imaging time point to insure that imaging data of the FITC-Dextran could be compared between imaging sessions. FITC-Dextran was cleared from the mice approximately 6 hours following its administration. Furthermore, we did not observe a decrease in vascular perfusion in non-irradiated mice (data not shown), thereby confirming that the phenomena of vascular dysfunction was induced by irradiation. The svOCT imaging showed that most of the larger vessels are patent and not structurally disrupted by irradiation. By day 14, vascular flow in vessels began to return, and indeed by day 20, vascular flow within tumor vessels was significantly increased and newly perfused vessels could be observed within the tumor (Figure 3B). Taken together, the direct comparison of spatially-co-registered fluorescence microangiography and svOCT imaging *in vivo* allowed us to distinguish between functional and dysfunctional vasculature following irradiation. This technique enabled us to monitor vascular response to irradiation at both structural and functional levels with spatial resolution of approximately 10 µm in the same animal. Furthermore, this method permitted quantification of functional vessel density and mean vessel density changes occurring over time following radiation treatment (Figure 3C, D). The data indicated that a single fraction of 30 Gy caused temporally-dynamic functional disruption in both large and capillary sized vessels, while leaving most of the larger vessels structurally intact. At day 20 after irradiation, most blood vessel perfusion resumed in vessels around the tumor periphery. Others have reported similar vascular perfusion kinetics in bulk subcutaneous xenograft tumors following single 10–20 Gy doses using Doppler ultrasound and immunohistochemistry [13]–[16]; however, the methods reported here provide the first opportunity to directly visualize and quantify these functional and structural changes in vessels *in vivo* within the tumor at high resolution (∼10 µm) in the same animal over time.

**Figure 4:**
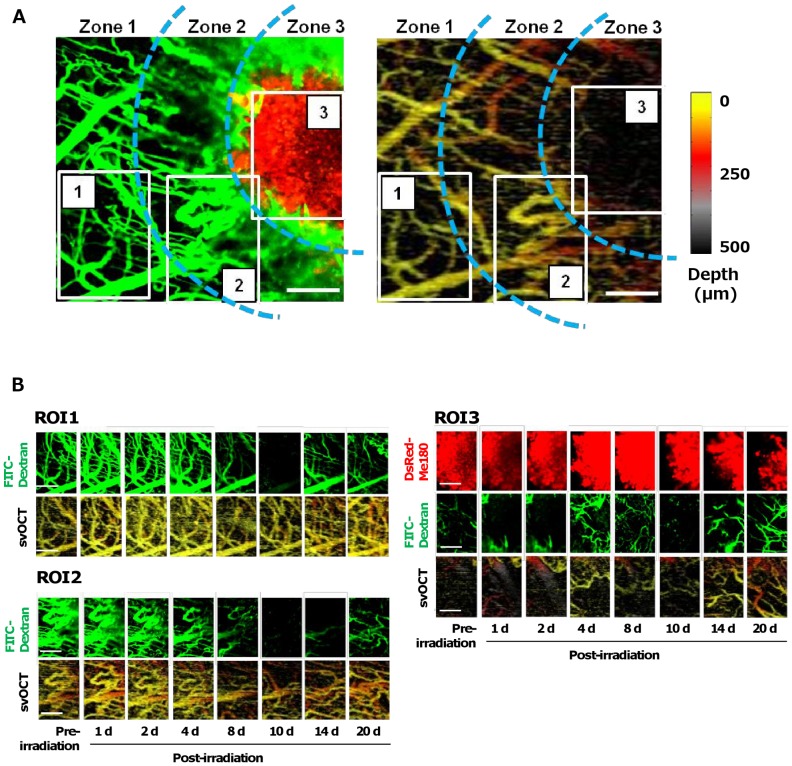
Longitudinal imaging of spatially-localized differences in tumor and vascular response to radiation. **A.** Examination of the temporally-specific changes in tumor size (DsRed, red), vascular perfusion (FITC-Dextran, green), and vessel patency (svOCT, color-coded for depth of vessels below the coverslip, inset) in 3 ROIs in spatially-distinct zones in the window chamber: 1–500 µm away from the tumor margin, 2 – adjacent to the tumor margin and 3 – within the tumor. **B.** Dynamic modulation of blood perfusion and vessel patency in large and small vessels in ROIs 1 (farthest from tumor) to 3 (inside the tumor). *Scale bars: 250 µm (A,B).*

To determine if the location of vessels with respect to the tumor made them differentially responsive to radiation, we examined the fluorescence and corresponding svOCT images in three spatially-distinct zones: 1 – approximately 500 µm away from the tumor margin, 2 – adjacent to the tumor margin and 3 – within the solid tumor. Figure 4 shows an example of temporally-specific changes in vascular perfusion (FITC-Dextran) and vessel patency (svOCT) in representative ROIs in these three different zones. Three ROIs of the same size were selected according to several vascular landmarks identified at each time point to allow for longitudinal co-registration (Figure 4A). We observed that vessels furthest from the tumor appear to be more radiation tolerant (maximal capillary occlusion seen at days 8 and 10) (Figure 4B, ROI 1), while vessels nearest the tumor boundary showed changes both functionally and structurally as early as day 4 (Figure 4B, ROI 2). They reached a more structurally and functionally stabilized state by day 20. In addition, vessels within the tumor were observed to have compromised functional and structural integrity even before irradiation (Figure 4B, ROI 3) [17] and, as described above, showed changes beginning as early as 2 days post irradiation. Meanwhile, after day 8, the tumor decreased in size in response to irradiation as indicated by DsRed fluorescence. These data suggest that vessels proximal to and within tumors are more radiosensitive compared with vessels further (∼0.5–1 mm) away, which themselves may not be normal given their proximity to the tumor edge [18]. These results demonstrate that intravital optical imaging can be used to study the radiosensitivity of vasculature within, at the periphery and at a distance from the tumor. Our data also demonstrate the ability to co-register multiple parameters including tumor cell location and structural and functional information about the microvasculature in a spatio- and temporally-specific manner. This could allow important morphological and functional based information to be obtained about the combined antitumor effects of radiation and emerging anti-vascular/anti-angiogenic therapies [19], [20].

### Imaging of Radiation-induced Vascular Thrombus Formation *In Vivo*

Vascular dysfunction can be caused by radiation-induced functional perturbation of interacting molecules on endothelial surfaces [21]. Hallmarks of this process are platelet adhesion and aggregation [22], increased expression of von Willebrand factor in extracellular matrix leading to recruitment of platelets [23], and accumulation of thrombin (a platelet agonist) [21], which are thought to be caused by radiation-induced endothelial injury. Platelet adhesion to denuded or damaged endothelium can lead to thrombus formation, which can cause occlusion of vascular lumen and impaired tissue perfusion [24], [25]. We investigated whether our experimental platform could be used to track spatiotemporal dynamics of platelet-derived thrombus formation and vascular function simultaneously in the microvasculature close to and within tumors following irradiation.

**Figure 5:**
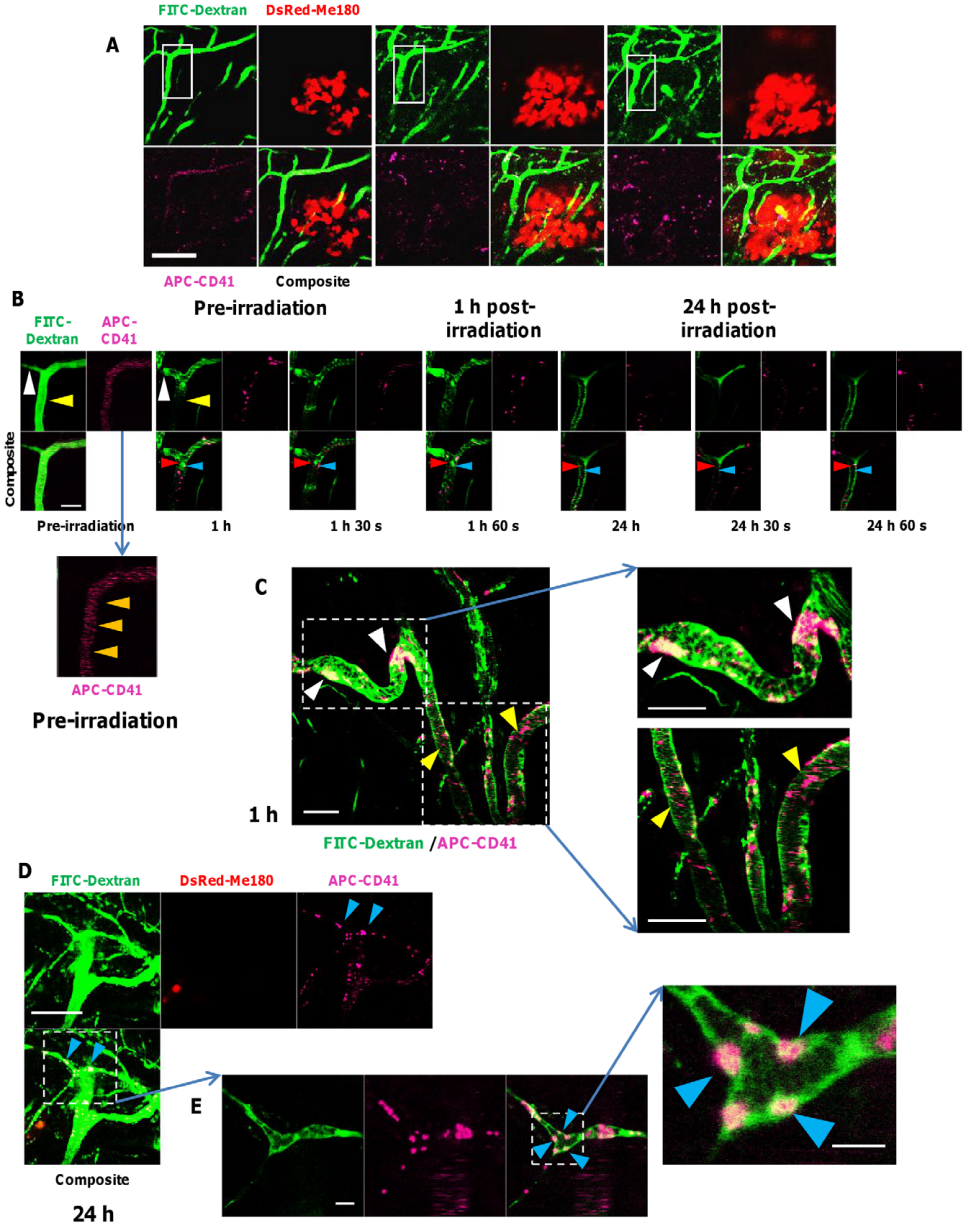
Real-time intravital optical tracking of radiation-induced platelet thrombus formation in tumor microvascular *in vivo*. **A.** FITC-Dextran (green) and APC-labeled anti-mouse CD41 antibodies (fuchsia) were used to visualize tumor microvasculature and platelets, respectively, in relation to the DsRed-Me180 tumor cells (red) before and (1 and 24 h) after a single fraction of 30 Gy. Blood flow is from top to bottom of images. **B.** Intravital fluorescence imaging (*inset of panel A*) taken pre-irradiation, 1 h and 24 h after irradiation, with serial images taken consecutively at 30 sec intervals, demonstrating transient formation of platelet thrombi. In pre-irradiation images, fast-moving blood cells, FITC-Dextran and APC-CD41 can be observed as horizontal lines (*orange arrows, inset of pre-irradiation images in panel B*) in the confocal fluorescence angiograms, as they pass out of the confocal scanning area faster than the image acquisition time (∼15 sec/channel). Compared to pre-irradiation, blood vessel dilation and blockage (*white arrow, B*), and impeded blood perfusion (*yellow arrow, B*) were seen 1 h after irradiation in some vessels when platelet thrombi form. Optical tracking of adhered FITC-Dextran-labeled RBCs (*red arrows, B*) and APC-CD41-labeled thrombotic plaques (*blue arrow, B*) formed early (1 h) after irradiation but clear after 24 h. After 24 h, early forming thrombi adhered to vessels walls were cleared in many cases. **C.** Microvascular function was compromised as early as 1 h after radiation by blockages caused mostly by larger (∼20–40 µm diameter) platelet thombi (*white arrow, C*), while microthrombi (∼2–5 µm diameter) transiently adhered to vessel walls. **D.** Typically, smaller vessels (∼12 µm diameter) were more prone at 1 and 24 h post-irradiation to thrombosis-induced occlusion (*blue arrows, D*) than larger (∼25 µm diameter) vessels where small thrombi forming at the vessel wall eventually cleared. **E.** Confocal fluorescence microscopy also revealed FITC-Dextran labeled RBCs aggregating with platelet thrombi to occlude capillaries, and in many cases adhering to microvessel walls (*blue arrows in inset, E*). *Scale bars: 125 µm (A), 25 µm (B), 50 µm (C), 125 µm (D), 25 µm (E) and 6 µm (inset, E).*

We used APC-labeled CD41 antibody to label platelets by injecting it intravenously along with FITC-Dextran repeatedly prior to each subsequent imaging session to visualize the vessels of the tumor area by confocal fluorescence microscopy. Imaging of the tumor area with confocal fluorescence microscopy was possible for up to 24 hours following irradiation (Figure 5A). Furthermore, serial imaging of a single capillary with a diameter of approximately 10 µm could be performed (Figure 5B). We observed that before irradiation, blood flow in vessels was uninterrupted and thrombi were not formed within tumor vessels (Figure 5B). Fast-moving blood cells, FITC-Dextran and APC-CD41 could be observed as horizontal lines in the confocal fluorescence angiograms prior to irradiation, as they passed out of the confocal scanning area faster than the image acquisition time. However, 1 hour following a single dose of 30 Gy, we observed the formation and sustained retention of multiple platelet-derived thrombi within capillary vessels (<20 µm diameter) resulting in localized microvascular occlusions with dramatically diminished blood flow. Figure 5C shows a representative area of diminished blood flow detected within the vessels caused by platelet thrombi. We observed multiple adhesion plaques composed of platelets with intravascular red blood cells (stained by FITC-Dextran) attached to the vessel wall (Figure 5D, E). Sustained adhesion of platelets to endothelial cells is believed to contribute to thrombosis and vascular occlusions following radiation exposure. A major molecular determinant of this response is thought to be significant and sustained up-regulated expression of PECAM-1 on irradiated vascular endothelial cells [26]. Persistent microvascular thrombi formation was observed over 24 hours after irradiation, resulting in impeded vascular perfusion; however this was a transient event, suggesting disintegration and clearance of some thrombi over time. These data demonstrate that it is possible to simultaneously visualize and track both blood flow and platelet thrombi formation and clearance, respectively, under dynamic conditions for up to 24 hours following irradiation by using intravenously administered FITC-Dextran and APC-labeled anti-CD41 antibody.

### Imaging of Perivascular and Stromal Elements of the Tumor *In Vivo*

**Figure 6:**
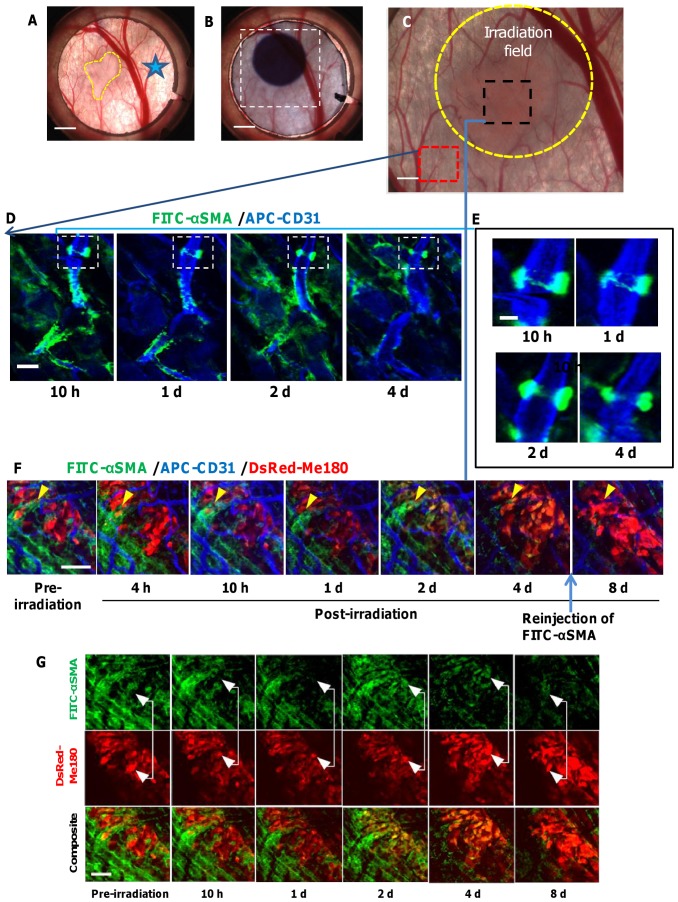
*In Vivo* fluorescence microscopic imaging of radiation response of perivascular cells. Photographs showing **A.** the tumor in the window chamber (*yellow outline*), the FITC-labeled α-SMA injection site (*star*), **B.** the location of the irradiation site (4 mm diameter) identified by radiochromic film, **C.** the ROIs (*red and black squares*) where intravital fluorescence imaging was performed relative to the tumor location and the radiation treatment field (*outlined in yellow*). A wide-field image of the tumor (*red*) was overlaid for visualization. **D.** Longitudinal fluorescence images showing FITC-labeled α-SMA+ pericytes (green) attached to capillaries stained with APC-CD31 (blue) in non-tumor tissue outside the radiation treatment field. **E.** Most non-tumor capillary pericytes appeared to be brightly fluorescent over the course of 4 days with little morphological alterations. **F.** Longitudinal fluorescence imaging of DsRed tumor tissue (red) (*same animal as in* ***D*** *and* ***E***) which received radiation treatment (30 Gy) revealed the presence of classically-appearing vessel-associated pericytes (*yellow arrow, F*). 4 days after radiation, pericytes coverage on tumor vessels was decreased and this continued to day 8, despite an additional injection of FITC-labeled α-SMA at day 8. **G.** Longitudinal fluorescence micrographs also show a significant increase in the co-localization of FITC labeled α-SMA (green) on the cell surface of DsRed-Me180 tumor cells (red) by days 2 and 4 after irradiation. This resulted in tumor cells appearing more yellow-orange in color due to increase in green FITC fluorescence mixed with the red DsRed tumor cell fluorescence. *Scale bars: 2.5 mm (A,B) 0.8 mm (C), 25 µm (D), 10 µm (E), 100 µm (F,G).*

Perivascular cells (pericytes and vascular smooth muscle cells) are important regulators of vascular formation, stabilization, remodelling and function [27]. Pericytes, which surround capillaries and contain contractile proteins, are thought to regulate blood flow [28]. Given their major role in maintaining vascular function, we sought to investigate the direct effects of ionizing radiation on perivascular coverage of tumor vasculature. To visualize the spatiotemporal changes in perivascular cells following irradiation, FITC-labeled antibody against alpha smooth muscle actin (α-SMA) was injected subcutaneously inside the DSWC once 15 minutes before fluorescence imaging to label the perivascular cells *in vivo*. α-SMA positive cells were identified as pericytes based on their characteristic morphology and if they were physically associated with blood vessels, while α-SMA positive cells that were not physically associated with blood vessels were determined to be myofibroblasts [29], [30]. Figure 6A–C shows the location of the antibody injection, the location of the irradiation site, the DsRed-Me180 tumor, and the ROIs where intravital fluorescence imaging was performed. In addition to the α-SMA antibody, APC-CD31 was used to label the vascular endothelial cells *in vivo*. There was no need for reinjection of the two agents for up to 4 days following irradiation, as the labeling of perivascular cells and endothelial cells persisted to enable imaging during this time. A small number of α-SMA positive pericytes were tracked on capillary-sized vessels in normal skin tissue outside the radiation treatment area, since they are known to be scarce in non-tumor-bearing mouse skin [29]. Staining of pericytes was confirmed since these cells were intimately associated with vessels and possessed all the morphological hallmarks of pericytes (Figure 6D, E). We observed no appreciable changes in α-SMA positive fluorescence in these cells over the course of 4 days. Importantly, the APC-labeled CD31 and FITC-labeled α-SMA agents retained the target-binding affinity *in vivo*, which allowed cell specific imaging within the tumor. In tumors, α-SMA positive cells were heterogeneously distributed on tumor microvasculature and within the stroma in the same animal. This observation is consistent with previous finding demonstrating that α-SMA is up-regulated in subcutaneously transplanted tumors [29]. We observed that there were no appreciable changes in the morphology or biodistribution of pericytes on tumor vasculature up to 2 days following irradiation (Figure 6F). However, there was a decrease in α-SMA fluorescence after 4 days. To exclude the cause of this as being the gradual clearance of unbound circulating α-SMA antibody from the first injection 4 days earlier, the same dose of FITC-labeled α-SMA antibody was administered subcutaneously within the DSWC area again prior to fluorescence imaging on day 8. There was no increase in FITC-labeled α-SMA fluorescence which we interpret as the depletion of pericyte antigen binding sites possibly caused by pericytes undergoing atrophy in response to capillary damage [31] or death and clearance from the vessels. This suggests that pericyte coverage of tumor vessels is reduced by day 4 following irradiation. Since pericytes in tumor vessels are critical for vessel integrity and function, pharmacological targeting of pericytes in tumors may be an attractive and efficacious approach for anti-angiogenic therapy [4]. The performance of the current imaging platform could enable such preclinical studies.

Unexpectedly we observed co-localization of FITC-labeled α-SMA on the cell surface of tumor cells at 2 and 4 days after irradiation (Figure 6G). One possible explanation is that ionizing radiation could induce morphologic and molecular alterations which are consistent with a change to a mesenchymal-like phenotype, a process known as epithelial-mesenchymal transition (EMT) [32], [33]. Emerging evidence shows that myofibroblasts can be derived from the epithelial (tumor) cells via EMT [34] and, while controversial, myofibroblasts have been implicated in the rapid development of fibrotic lesions composed of proliferating myofibroblasts and fibroblasts underlying the pathogenesis of irradiation-induced fibrosis [35]. Further studies are needed to investigate this observation in more detail.

### Spatially-localized Quantitative Profiling of Gene Expression

**Figure 7:**
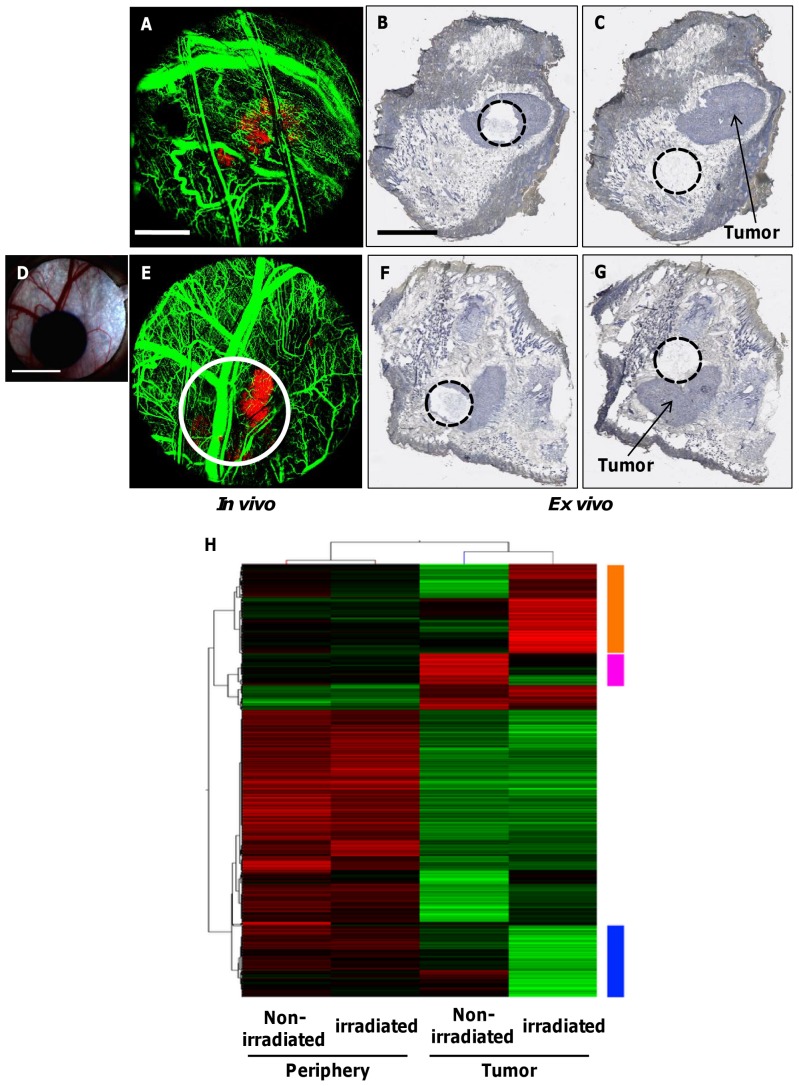
Spatially-localized analysis of genomic expression in non-irradiated and irradiated tumors. **A.** *In vivo* fluorescence imaging of DSWC area showing DsRed-Me180 tumor (red) and FITC-Dextran labeled vasculature (green) in a non-irradiated mouse. **B, C.** Corresponding *ex vivo* tissue cross sections (counterstained with hematoxylin for histological context) of non-irradiated mouse showing precise location of LCM capture sites within and outside (periphery) the tumor, respectively. **D.** Photograph of radiochromic film showing the irradiation field (single 30 Gy dose) within the window chamber area of another mouse. **E.** *In vivo* fluorescence imaging shows location of radiation field (*white circle*) with respect to tumor and vasculature in the irradiated mouse. **F, G.** Corresponding *ex vivo* tissue cross sections of irradiated mouse showing locations of LCM capture sites within and outside the irradiated tumor, respectively. The mouse was sacrificed 4 days following irradiation for gene expression analysis. **H.** Two-way hierarchical clustering of probes showing the highest variability in 4 samples of irradiated and non-irradiated tumor and peripheral tissue areas. Orange: probes showing specific up-regulation in the irradiated tumor. Blue: probes showing specific down-regulation in the irradiated tumor. Pink: probes showing specific up-regulation in the non-irradiated tumor. (n = 2 mice) *Scale bars: 2 mm (A), 3 mm (B), and 4 mm (D).*

The analysis of gene expression in irradiated tumors can be a valuable tool for the assessment of transcriptional changes during the various stages of radiation response *in vivo* [36]. To correlate such gene expression changes at 4 days following irradiation with our imaging data, we obtained samples by laser capture microdissection (LCM) in a spatially-localized manner from tumor and non-tumor (periphery) tissue areas within the window chamber (Figure 7A–G). RNA isolated from these defined regions (3×3 mm^2^) was subjected to whole genome microarray analysis using the Illumina platform. RNA isolated from the LCM samples using the window chamber model was limited and showed lower integrity than that seen in normal tissue (RNA integrity numbers, RIN, of 4.5–7.0). To address this, we utilized the NuGen Ovation Pico amplification kit, which uses a random priming strategy to allow as little as ∼10 ng of total RNA to be profiled.

After normalization and filtering, 34013 probes on the Illumina Mouse Whole Genome microarray were subjected to further analysis. Two-way hierarchical clustering (Figure 7H) showed that samples grouped based on tumor *verses* periphery sections grouped together the strongest. We performed manual extraction of probes to find genes specific to the irradiated tumor samples. 373 probes were down-regulated and 471 were up-regulated in the irradiated tumor (blue and orange bars in Figure 7H, respectively) compared to non-irradiated tumor. These probes overlapped >99% with a list of 3689 probes that showed a 2 fold difference between the irradiated tumor and all other samples. Gene ontology (GO) enrichment (FDR p<0.1) of the up-regulated genes in the irradiated tumor showed a number of different categories (Table S1) related to the immune system. This may represent a local response of immune system infiltration into the tumor microenvironment [37], [38]. Genes involved in angiogenesis that were found up-regulated included Vegfa, Mmp9 and Mmp2 [39]. Down-regulated genes in the irradiated tumor in significantly enriched GO categories (Table S2) related to smooth muscle and pericytes such as Des, Myh4, Ttn and Mybpc2. A number of eukaryotic initiation factors (Eif2b3, Eif3a, Eif3d, Eif4a2, Eif4e) were also down-regulated, suggesting translational machinery dysregulation. In the non-irradiated tumor, we found 165 probes (pink bar in Figure 7H) that were up-regulated. As expected, many of the genes in this list were markers of squamous cell carcinoma (the tumor of origin for the cell line used in this study) such as Cdh11, Postn, and Tnc [40]. These data demonstrate the feasibility of obtaining *in vivo* optical imaging and *ex vivo* gene expression data sets in a spatially- and temporally co-registered manner at any one time point after irradiation within a tumor or other area in the window chamber.

## Discussion

Our overall objective was to develop a robust method of studying complex tumor and vascular response to ionizing radiation evolving over time *within the living tumor* with exquisite spatial and temporal control and in real-time. We have introduced a new preclinical experimental platform for cellular-level, non-invasive longitudinal optical imaging to study localized radiobiological response of tumor cells as well as their vascular and perivascular compartments *in vivo*. We combined spatially-targeted focal irradiation with intravital multimodal optical microscopy (e.g. fluorescence and svOCT) in a tumor-bearing DSWC mouse model. Our platform includes some techniques, such as the small animal irradiator and intravital fluorescence microscopy, which are commercially available. Additionally, we have introduced new complementary technical capabilities, such as svOCT imaging and the custom built mouse holder for immobilizing the subject during x-ray irradiation and microscopy which, in combination, provide an important advancement to study radiobiological response phenomena *in vivo* in murine models of cancer.

This platform circumvents several previous technical limitations in preclinical radiobiology studies:

- The microirradiator enables precise focal treatment of small tumors in the window chamber with a controlled dose and beam profile. The precise location of the irradiation beam can be identified and registered to subsequent longitudinal optical imaging. Furthermore, the microirradiator provides for image-guided delivery of the radiation beam at sub-millimeter resolutions which is currently not possible with conventional techniques (e.g. use of lead shielding).
- The transparent window chamber model permits simultaneous visualization of multiple tumor components (e.g. tumor cells, vasculature, perivascular cells, and platelets) using multiplexed confocal fluorescence microscopy with single-cell resolution and svOCT imaging of microvascular structural patency.

A key advantage of our approach is that longitudinal imaging allows us to track dynamic changes in tumor vascular networks or individual tumor vessels and to compare how different vessel types respond in the same animal over time. By using multiple molecularly-targeted fluorescent reporter probes *in vivo*, we were able to track radiation-induced (vessel size-dependent) structural, functional and cellular alterations in real-time. We have demonstrated the ability to quantify these changes for up to 3 weeks after treatment in the same animal. Indeed, the DSWC allowed us to compare irradiated *verses* non-irradiated tissues in the same mouse, providing an internal control comparison. Furthermore, we could track the temporal profiles of perivascular cell response and radiation-induced microvascular thrombogenesis in irradiated tumor vasculature. Analysis of tumor cells, microvasculature and pericytes at high spatial resolution could also provide information relevant to cell-to-cell interactions resulting from irradiation *in vivo*.

Our results reveal progressive distortions of tumor vascular geometry and reduced microcirculatory perfusion caused by radiation-induced thrombi, which could contribute to transient deprivation of oxygen and nutrients in the tumors. Such localized inhibition of microvascular function could influence tumor hypoxia status at a microscopic level [16]. This in turn could induce radioresistance due to hypoxia or select phenotypically for radiation-resistant tumor cells [19], [41]. Our platform could be used in the future to study these phenomena in preclinical tumor models using clinically-relevant radiation treatment regimens [42]. We recognize that using APC-CD41 intravascularly to image platelet thrombosis could be problematic due to microvascular occlusions seen at later time points (>4 days) that reduce the delivery efficiency of the fluorescence antibody through the irradiated tumor vasculature. As an alternative strategy, the use of transgenic mice with GFP-expressing platelets could overcome this limitation [43]. Furthermore, the platform could allow important morphological and functional based information to be obtained about the combined antitumor effects of radiation and emerging anti-vascular/anti-angiogenic therapies [19], [20]. We have confirmed that the *in vivo* performance of the platform allows for irradiation of sub-millimeter tissue volumes and imaging of multiple tissue components simultaneously at single-cell resolution. Multispectral fluorescence and svOCT microscopic imaging were found to be sensitive to acute tissue, vascular and cellular changes that occur over relatively short periods of time (e.g. 1 hour) as well as delayed changes following irradiation. In addition, fluorescence labeling using ligand-specific antibody provided for cell type-specific imaging *in vivo* so that different tumor components (e.g. cells, vessels, pericytes) can be identified and studied over time.

Laser capture microdissection of specific regions from within or outside the tumor or area of irradiation allowed for the determination of the effects of treatment on the transcriptomic response both in normal and tumor tissue. The results from these microarrays provided a *proof-of-concept* that spatially-localized extraction and accurate genomic profiling of the material can be readily accomplished in concert with the imaging methodologies presented here to give a unique and multi-faceted view of the spatio-temporal effects of irradiation. This technique enables high spatial resolution mappings of radiation response throughout the tissue moving outwards from the tumor and area of irradiation, allowing for assessment of not only direct, but also indirect effects of irradiation. For *ex vivo* analysis of tissues the mouse must be sacrificed; but this genomic level data can be compared with the serial *in vivo* optical imaging data obtained longitudinally prior to euthanasia. Although it would have been ideal to further separate the tissue collected into tumor cells, endothelial cells, immune cells, etc. in order to more accurately identify gene expression responses in each cellular compartment, this was not possible with the current technique. Further refinement of our amplification techniques is needed to work with the RNA isolated from the DSWC samples which is of only moderate integrity and thus more difficult to profile.

The experimental platform is also flexible enough to be applied to more clinically-relevant orthotopic tumors grown in transparent windows e.g. cranial, pancreatic and spinal cord, and to permit future addition of emerging imaging technologies that offer different/complementary advantages to studying tumor response. For example, emerging photoacoustic microscopy technology would allow longitudinal observation of tumor microvasculature and measure oxygenation dynamically *in vivo* [44]. In addition, the flexibility in the pattern of irradiation would allow more complex dependencies, such as the bystander effect, to be studied directly *in vivo* [45].

In conclusion, our preclinical experimental platform provides a powerful way to study the complex radiobiological changes in solid tumor compartments (cellular, vascular, perivascular) in a multiplexed manner *in vivo*, including studying the modulatory effects from genes either driving or influenced by radiation response. Our goal at this stage is to report the development and demonstrate the utility of this novel methodology where we have consolidated existing and new technical capabilities to address barriers that have previously limited advancement in the preclinical study of radiation response in tumors. Our approach compliments existing radiobiological research tools to possibly unlock new frontiers in our understanding of the biomolecular actions of ionizing radiation in solid tumors to improve the clinical impact of radiation oncology.

## Methods

### Animal Studies

All animal procedures were conducted in accordance with appropriate regulatory standards under protocols AUP#1566.5 and #2407.1 approved by the University Health Network Institutional Animal Care and Use Committee, following institutional guidelines for the proper and humane use of animals in research. 4–6 weeks old female athymic nude mice (strain NCRNU-F) were ordered from Taconic, Hudson, New York.

### Anaesthetics

During experimental procedures, mice were anaesthetized using a mixture of ketamine and xylazene (80 mg/kg and 5 mg/kg respectively), diluted in saline and administered by intraperitoneal injection.

### Cell Culture

DsRed fluorescent Me180 human cervical cancer cells (provided by Dr. Richard Hill, OCI) were grown in MEM-α medium (GIBCO BRL, Gaithersburg, MD) supplemented with 2 mM glutamine, 50 units/ml penicillin, 50 units/ml streptomycin, 10% heat-inactivated fetal bovine serum (GIBCO BRL, Gaithersburg, MD) and 400 µg/ml Geneticin (G418, Invitrogen, Burlington, ON) at a temperature of 37°C in an environment containing 5% CO_2_. Before use, the cells were trypsinized, counted and suspended in 10% phosphate buffered saline (PBS).

### Dorsal Skinfold Window Chamber (DSWC) Tumor Model

Window chambers were surgically implanted on the dorsal skinfold of anesthetized mice using established methods [46]. At the time of surgery, prior to placement of a standard 12 mm diameter quartz glass microscope coverslip (Warner instruments, Hamden, CT), approximately 500,000 DsRed-Me180 cells suspended in 5 µL PBS were injected directly in the retractor muscle (fascia) of the mouse dorsal skinfold. Immediately after injection of the cells, the coverslip was placed on the tissue and secured using a plastic o-ring device. After surgery, animals were housed at 33°C and 50% humidity with free access to food and water and maintained under standard 12 h light/12 h dark cycles. Tumors were grown until they reached 3–5 mm diameter in the window chamber, which developed after approximately 7–10 days. To improve imaging quality, prior to each imaging session, the coverslip was carefully and quickly removed, the tissue was irrigated gently with warm phosphate-buffered saline using a needle syringe to gently clean any cellular debris on the tissue surface, and a new coverslip was put in place.

In order to avoid motion of the animal during x-ray irradiation and imaging, a custom mouse restrainer was built. The window chamber was fastened onto a rectangular (4×8 cm^2^) metal plate, which was then placed onto the custom-built restrainer. The microscope objective was above the glass coverslip for imaging. A built in resistive heating element (Harvard Appartus, Massachusetts, USA) was built into the base of the plastic restrainer to maintain physiological temperature of the animal during all experimental procedures.

### Wide-field Fluorescence Microscopy and X-ray Micro-irradiation

White light reflectance, transmission and fluorescence images of DSWC were obtained using a stereomicroscope (Leica Microsystems, Concord, Ontario) prior to irradiation. Images were acquired prior to and post irradiation for up to 20 days. Irradiation was performed using an image-guided small animal irradiation system (XRad225Cx, Precision X-Ray, Inc., North Brantford, CT). The system consists of a dual focal spot, 225 kVp x-ray tube, flat-panel amorphous silicon imager mounted on a c-arm gantry, and mechanical translation stage for animal positioning. To deliver ionizing radiation dose to the tumor in the window chamber, a photograph of the window chamber was first taken with the stereomicroscope (Figure 1C, D). A digital grid, consisting of 1×1 mm^2^ squares, was overlaid and centred onto the white light reflectance image of the window chamber to map the location of the fluorescent tumor in the chamber for irradiation (Figure 1E). The grid image acted as a guide for the operator to move the irradiator stage in x and y directions toward the target region. X–Y co-ordinates recorded from the grid image allowed precise positioning of the mouse using the stage micrometer on the micro-irradiator system. Irradiations were performed using 100 kVp x-rays (HVL 3.1 mm Al). Custom brass collimators were employed to create a beam of 2.5 mm in diameter. Dosimetry was performed using Gafchromic EBT film (ISP Inc., Wayne, NJ) which consists of a radiosensitive monomer that polymerizes upon irradiation [47]. To perform dosimetry, films were sandwiched between the window chamber frames and irradiated by the micro-irradiator. Two additional films were used to account for backscatter, such that the total thickness of the stack was similar to that of the average dorsal skin after surgery. The glass cover slip was present during film irradiation to mimic the setup of the DSWC. One day was given for the films to stabilize, and the top film was then scanned with a flat-bed scanner (Epson Inc., Markham, ON). Based on the net optical density difference before and after irradiation, the dose was determined by a dose-net optical density calibration curve [48], [49]. To maximize the dose rate, the animal was set up as close as possible to the x-ray source (approximately 3.5 cm distance from the collimator) and was irradiated using the maximum current of the system. The resulting dose rate was 2.33 Gy/min. The Gafchromic film verified the targeting accuracy of dose delivering dose to the desired location. Immediately following irradiation, a white light digital photo containing the irradiated radiochromic film confirmed the location of radiation delivery.

### Speckle Variance Optical Coherence Tomography (svOCT) Imaging

Measurements were performed using a 36 kHz Fourier domain mode locking (FDML) swept-source OCT system similar to that described previously [50]. Briefly, the system used a swept laser source incorporating a polygon-based tunable filter with a sweeping range of 112 nm centered at 1310 nm, with an axial resolution of 8 µm in tissue and average output power of 48 mW. The imaging scan area was 6×6 mm^2^ with an imaging depth in tissue of ∼2 mm. The mouse was secured in the custom frame to reduce bulk tissue motion and to maintain body temperature during imaging. Inter-frame speckle variance images were calculated from a set of n = 8 B-mode structural intensity images acquired from the same location. The difference in the viscosity properties of fluids and solids led to a difference in the magnitude of the calculated variance, providing contrast between solids (tissue) and liquids (blood vessels) in svOCT images [12].

Post processing of svOCT images was performed using MATLAB (The MathWorks, Massachusetts, USA). Shadowing artefacts beneath blood vessels occur in svOCT images due to the forward scattering of photons by blood. A step down exponential filter was applied to reduce these artefacts [51]. Moving downwards from the surface of the tissue, the filter attenuated the speckle variance signal of each pixel by a factor proportional to the sum of the filtered speckle variance pixels above it. Images were medial filtered and a hard threshold was applied to reduce noise. Each z-slice in the 3-D dataset was then encoded with a RGB color dependent on its depth in the stack. The transparency (alpha channel) for each pixel in the stack was set to its speckle variance intensity. The RGB and alpha channels were then combined into a stack of PNG images, which were layered axially using Amira™ software (Visage Imaging, Version 5.2.1, San Diego, CA, USA).

To quantitatively measure the vascular changes following radiation therapy, an established algorithm was implemented to calculate vascular density [52], [53]. Regions of interest (ROIs) were manually selected from each time point image. The ROI image was binarized using a standard histogram shape-based image thresholding script in Matlab (MathWorks Inc., Natick, MA). Vascular density was calculated by dividing the number of pixels that contained blood vessels (based on non-zero svOCT intensity levels) by the total number of imaged pixels in the ROI. The vascular density difference was then calculated by subtracting the pre-irradiation density from the density at each time point after irradiation.

### Intravital Confocal Fluorescence Microscopy and Optical Probes

Intravital confocal fluorescence microscopy was performed on the DSWC using LSM 510 Meta (Carl Zeiss, Jena, Germany) to simultaneously visualize multiple fluorescently labeled tissue components. Imaging parameters were optimized for maximum fluorescence contrast for each labeling agent prior to all experiments. This allowed the maximum signal-to-noise images to be obtained by varying the excitation laser power, confocal pinhole diameter, and detector gain settings on the photomultiplier tube. Confocal image scans were obtained at 1024×1024 pixels and averaged four times per scan. In tumor-bearing mice, several regions-of-interest (ROIs) were imaged in the tumor and surrounding tissue. All mice were serially imaged prior to and over multiple days up to 20 days after irradiation using the same confocal imaging settings between time points for each experiment. This enabled direct comparison of fluorescence intensity between images over time. For intravital confocal fluorescence microscopy, we used the following long working distance objectives: 5× Fluars, 10× EC Plan-Neofluar, and a 40× Water Plan-Apochromat (Carl Zeiss, Jena, Germany). On occasion, to visualize microscopic details *in vivo*, a digital zoom was used with the 40× Water Plan-Apochromat. Prior to each imaging time point, window chamber-bearing mice received intravenous or intradermal injections of fluorescent probes using a 30 gauge butterfly needle. FITC-Dextran (3 mg/ml) (Sigma-Aldrich, Oakville, ON) with a molecular weight of 2.5 MDa (488 nm excitation, 500–550 nm emission) was used to visualize the vasculature. Allophycocyanin (APC)-conjugated rat anti-mouse CD31 (platelet endothelial cell adhesion molecule PECAM-1) monoclonal antibody (0.03 mg/ml) (BD Biosciences, Mississauga, Ontario) was used to visualize vascular endothelial cells (633 nm excitation, 650–710 nm emission). Platelets were labeled using APC-conjugated rat anti-mouse CD41 monoclonal antibody (0.5 mg/ml) (BD Biosciences, Mississauga, ON) (note, in Figures, CD41 fluorescence is pseudo-colored fuchsia for better visualization). Pericytes were labeled using FITC-αSMA (2.3 mg/ml) (Sigma-Aldrich, Oakville, ON). Confocal images were acquired from the same tissue imaging plane at each time point, using stationary vascular landmarks.

### Histological Preparation and Laser Capture Microdissection

For RNA extraction and quantification, we used laser capture microdissection (LCM) to localize specific ROIs of *ex vivo* frozen tissue samples either untreated or treated by ionizing radiation. For this, tumor-bearing dorsal skinfold tissue was surgically resected from anaesthetized mice with the metal chamber mount intact either without radiation treatment or 4 days following radiation treatment at 30 Gy. The metal chamber was swabbed down with RNaseZap solution (Ambion Inc. Streetsville, Ontario) and immediately removed from the dorsal skinfold. The window chamber tissue and surrounding skin was surgically resected and immediately frozen in liquid nitrogen with Tissue Tek Optimum Cutting Temperature (O.C.T.) compound (Sakura Finetek USA, Inc., Torrance, CA). Frozen tissue sections (10 µm thick) were cut as a cross section through the lateral plane of the tissue to reveal an approximately 1 cm diameter disk of tissue. The tissues were placed on standard glass microscope slides pretreated with RNaseZap solution and were stored at −80°C. RNase-free technique was used for the entire procedure. LCM was carried out using the Pixel IIe laser capture microdissection system (Arcturus-Life Technologies, Carlsbad CA) with RNase Zap sprayed on all contact surfaces to minimize the potential of RNase contamination during tissue handling and processing. To achieve spatial co-localization of *in vivo* optical imaging in tissues treated with radiation with genetic expression profiles, two areas within and outside of tumor regions, each measuring approximately 3×3 mm^2^, were captured from irradiated and non-irradiated mice across six serial *ex vivo* tissue sections counter-stained with Arcturus Staining Solution (Applied Biosystems, Foster City, CA) (the tumor was approximately 3 mm in diameter and the radiation treatment beam was 4 mm in diameter and centered on the tumor). Each LCM sample contained approximately 2000 cells captured and immediately placed in extraction buffer for 30 min at 37°C. Samples were sent for RNA isolation and microarray analysis. Serial sections of the tissue (8 µm thick) were also cut and used for standard histological staining (H&E) allowing co-registration of the LCM samples with specific histological landmarks and correlation with histopathology. We used the same digital grid overlaid on images of the whole DSWC tissue area to plan the LCM procedures and to spatially co-register the LCM samples with the radiation treated tumor location.

### RNA Extraction

The Arcturus PicoPure RNA Isolation Kit (Life Technologies, Carlsbad CA) was used for the extraction of RNA from LCM samples following manufacturer's instructions. Briefly, 30–50 µl of Extraction Buffer was pipetted into the bottom of the LCM tube. The tube was inverted via a swinging motion to transfer to liquid to the top of the lid containing the LCM material. The sample was then incubated in a dry oven at 42°C for 30 minutes keeping the extraction buffer on the lid. The purification column was preconditioned with 250 µl of conditioning buffer and incubated at room temperature for 5 minutes. The LCM tube was centrifuged for 2 minutes at 800×g to collect the tissue extract at the bottom of the tube. A 1∶1 volume of 70% Ethanol to Extraction Buffer was added to the tissue extract and mixed well by pipette. The resultant mixture was added to the preconditioned column. RNA was bound to the column by centrifugation for 2 minutes at 1,000×g followed by centrifugation at 16,000×g for 30 seconds to remove the flow through. The column was washed with 100 µl of Wash Buffer 1 and centrifuged for 1 minute at 8000×g. DNase1 was added to the column and incubated at room temperature for 15 minutes. 40 µl of Wash Buffer 1 was added to the column and mixed well then centrifuged at 8,000×g for 15 seconds. 100 µl of Wash Buffer 2 was added to the column and centrifuged for 1 minute at 8000×g. A second 100 µl of Wash Buffer 2 was added and centrifuged for 2 minutes at 16000×g. The extraction column was transferred to a new 0.5 ml tube and the RNA was eluted with 15 µl Elution Buffer pre-warmed to 42°C. The column was incubated at room temperature for 1 minute, centrifuged briefly at 1,000 g (1 min) before the final elution step of a 16,000×g centrifugation (2 minutes). RNA quantity was determined by NanoDrop (Thermo; Ontario, Canada) was then screened using Agilent's Bioanalyzer (Santa Clara, CA). All samples were stored at −80°C until use.

Gene expression profiling was carried out using Illumina Whole Genome arrays by the UHN Microarray Centre (www.microarrays.ca). 25 ng of total RNA were subjected to WT-Ovation Pico RNA Amplification (NuGen Technologies Inc., San Carlos, CA) to produce 4 µg of cDNA. This was followed by hybridization following the details of the Illumina Solution Application Note#2, in which 1.5 µg of cDNA was hybridized to Illumina WG-6 v2 mouse whole genome expression microarrays (Illumina Inc., San Diego, CA). Arrays were hybridized overnight and washed and scanned as per manufacturer's instructions. The resultant images were quantified and subjected to quality control checks to confirm that all arrays passed the necessary quality cut-offs. Data was then subjected to informatics analysis.

### Informatics Analysis

Microarray data from the Illumina Mouse WG-6-v2-r2 chip were quantified in GenomeStudio (v2011.1, Illumina). Intensity reads for each of the 45281 probes were imported into R (v2.13.1) using the Bioconductor LUMI package and a number of quality control metrics were generated [54]. All samples passed for further analysis. Normalization and analysis was conducted in GeneSpring (v11.5.1, Agilent). Normalization consisted of a per-array shift to the 75th percentile followed by a probe-based transformation to the median of all samples and logging of the data (base 2). Due to the low sample size (n = 1 for each of 4 categories) a strict pre-filtering was conducted such that only probes in the upper 50th percentile of measured expression on any one of the 4 arrays was considered for further analysis. A cutoff of a standard deviation greater than 1 for each probe across all arrays was used to find probes that varied the most between samples. Two-way hierarchical clustering was performed using a Pearson centered distance metric under average linkage rules. Gene ontology enrichment defining categories of extracted gene sets used a hypergeometric test with a Benjamini-Yekutieli multiple testing correction of p<0.1 [55].

### Statistical Analysis

Numeric data were analyzed for statistical significance using two-tailed paired Student's t test. P<0.05 was considered significant.

## Supporting Information

Table S1 **Significant gene ontology categories up-regulated in the irradiated tumor.**
(PDF)

Table S2 **Significant gene ontology categories down-regulated in the irradiated tumor.**
(PDF)
