# Supplementary material for: In Vivo Optical Imaging of Tumor and Microvascular Response to Ionizing Radiation
Source: PLoS One. 2012 Aug 22;7(8):e42133. doi: 10.1371/journal.pone.0042133 (PMC3425534; doi:10.1371/journal.pone.0042133)
Supplement: Table S1 — Significant gene ontology categories up-regulated in the irradiated tumor. (PDF) [file pone.0042133.s001.pdf]

GO ID	GO ACCESSION	GO Term	p-value	corrected p-value
1562	GO:0002376	immune system process	4.0243056E-5	0.05097219
1664	GO:0002478	antigen processing and presentation of exogenous peptide antigen	7.954504E-8	0.00130978
1681	GO:0002495	antigen processing and presentation of peptide antigen via MHC class II	4.258328E-6	0.00876466
1690	GO:0002504	antigen processing and presentation of peptide or polysaccharide antigen via MHC class II	9.003556E-6	0.01347743
3841	GO:0005578	proteinaceous extracellular matrix	1.1463071E-6	0.00471875
4964	GO:0006955	immune response	7.616064E-7	0.00418018
7096	GO:0009897	external side of plasma membrane	6.709349E-6	0.01104755
11793	GO:0019882|GO:0030333	antigen processing and presentation	6.249866E-5	0.06756376
11795	GO:0019884	antigen processing and presentation of exogenous antigen	4.1456892E-7	0.00341313
11797	GO:0019886|GO:0042591|GO:0048005	antigen processing and presentation of exogenous peptide antigen via MHC class II	4.258328E-6	0.00876466
13496	GO:0031012	extracellular matrix	1.4476072E-6	0.00476723
18599	GO:0042605|GO:0042606|GO:0042607	peptide antigen binding	6.690673E-5	0.06756376
19421	GO:0043487	regulation of RNA stability	5.7050995E-5	0.06709975
19422	GO:0043488	regulation of mRNA stability	3.861347E-5	0.05097219
23156	GO:0048002	antigen processing and presentation of peptide antigen	1.9227598E-6	0.00527666
