# Supplementary material for: In Vivo Optical Imaging of Tumor and Microvascular Response to Ionizing Radiation
Source: PLoS One. 2012 Aug 22;7(8):e42133. doi: 10.1371/journal.pone.0042133 (PMC3425534; doi:10.1371/journal.pone.0042133)
Supplement: Table S2 — Significant gene ontology categories down-regulated in the irradiated tumor. (PDF) [file pone.0042133.s002.pdf]

GO ID	GO ACCESSION	GO Term	p-value	corrected p-value
2597	GO:0003779	actin binding	1.5735297E-4	0.05435988
2707	GO:0003954	NADH dehydrogenase activity	1.7183235E-4	0.05441516
3880	GO:0005622	intracellular	2.5225436E-12	2.8757835E-8
3890	GO:0005634	nucleus	1.512274E-4	0.05387633
3962	GO:0005737	cytoplasm	1.487151E-7	2.0014567E-4
4543	GO:0006457|GO:0007022|GO:0007024|GO:0007025	protein folding	1.5081478E-4	0.05387633
4914	GO:0006891	intra-Golgi vesicle-mediated transport	2.0809889E-4	0.06411882
5008	GO:0007010	cytoskeleton organization	7.057107E-5	0.03027607
5603	GO:0008092	cytoskeletal protein binding	1.5548325E-5	0.01042683
5637	GO:0008137	NADH dehydrogenase (ubiquinone) activity	1.7183235E-4	0.05441516
8864	GO:0015629	actin cytoskeleton	7.748297E-5	0.03038066
12559	GO:0022900	electron transport chain	5.533425E-5	0.02623894
12637	GO:0030016	myofibril	2.9249799E-8	1.1115247E-4
12638	GO:0030017	sarcomere	1.355918E-7	2.0014567E-4
12639	GO:0030018	Z disc	6.829969E-6	0.00598953
12645	GO:0030029	actin filament-based process	7.4360105E-5	0.03027607
12652	GO:0030036	actin cytoskeleton organization	3.5611058E-5	0.01911249
14122	GO:0031674	I band	8.5683473E-7	9.7682E-4
19135	GO:0043170|GO:0043283	macromolecule metabolic process	3.6882684E-5	0.01911249
19189	GO:0043226	organelle	8.155504E-8	1.5495907E-4
19190	GO:0043227	membrane-bounded organelle	1.2133692E-5	0.00922187
19191	GO:0043228	non-membrane-bounded organelle	2.7885251E-5	0.01673164
19192	GO:0043229	intracellular organelle	7.8970245E-8	1.5495907E-4
19194	GO:0043231	intracellular membrane-bounded organelle	1.159504E-5	0.00922187
19195	GO:0043232	intracellular non-membrane-bounded organelle	2.7885251E-5	0.01673164
19251	GO:0043292	contractile fiber	3.9511264E-8	1.12610374E-4
20156	GO:0044237	cellular metabolic process	5.7539855E-5	0.02623894
20179	GO:0044260|GO:0034960	cellular macromolecule metabolic process	2.4623855E-6	0.002552
20186	GO:0044267	cellular protein metabolic process	7.359119E-5	0.03027607
20285	GO:0044422	organelle part	6.0881366E-6	0.0057839
20287	GO:0044424	intracellular part	8.71548E-11	4.967968E-7
20307	GO:0044444	cytoplasmic part	7.994678E-5	0.03038066
20312	GO:0044449	contractile fiber part	1.5800515E-7	2.0014567E-4
23307	GO:0048193	Golgi vesicle transport	4.527842E-5	0.022443
24130	GO:0050136	NADH dehydrogenase (quinone) activity	1.7183235E-4	0.05441516
25053	GO:0051082	unfolded protein binding	1.3541494E-5	0.0096486
